# Genetic differentiation and genetic structure of mixed-ploidy *Camellia hainanica* populations

**DOI:** 10.7717/peerj.14756

**Published:** 2023-02-22

**Authors:** Hailang Tong, Hongda Deng, Zhiqiang Han

**Affiliations:** 1 Central South University of Forestry and Technology, The College of Forestry, Changsha, China; 2 Central South University of Forestry and Technology, The Laboratory of Forestry Genetics, Changsha, China

**Keywords:** *Camellia hainanica*, Polyploidy, Genetic differentiation, Genetic diversity, Genetic structure

## Abstract

*Camellia hainanica*, which is common in China’s Hainan Province, is an important woody olive tree species. Due to many years of geographic isolation, *C. hainanica* has not received the attention it deserves, which limits the exploitation of germplasm resources. Therefore, it is necessary to study population genetic characteristics for further utilization and conservation of *C. hainanica*. In this study, 96 individuals in six wild* Camellia hainanica* populations were used for ploidy analysis of the chromosome number, and the genetic diversity and population structure were investigated using 12 pairs of SSR primers. The results show complex ploidy differentiation in *C. hainanica* species. The ploidy of wild *C. hainanica* includes tetraploid, pentaploid, hexaploid, heptaploid, octoploid and decaploid species. Genetic analysis shows that genetic diversity and genetic differentiation among populations are low. Populations can be divided into two clusters based on their genetic structure, which matches their geographic location. Finally, to further maintain the genetic diversity of *C. hainanica*, ex-situ cultivation and in-situ management measures should be considered to protect it in the future.

## Introduction

*C. oleifera* is the most important woody oil crop in China, and has excellent potential for development. This tree species plays an essential role in ensuring national grain and oil safety, socialist economic construction and ecological civilization construction (Lin et al., 2022; Gong et al., 2020). *C. hainanica* is a new species of the *Camellia* Sect. *Oleifera* was discovered in Hainan Province in 2020, and is an essential genetic resource for breeding. Due to its long-term geographical isolation, *C. hainanica* has not received due attention. The research and utilization of *C. hainanica* remain in its infancy, which means that the systematic evaluation of this resource has not been carried out domestically and overseas.

Species genetic structure and genetic differentiation are an important part of biodiversity conservation. Genetic variation underlies the evolutionary potential of species and is critical to the adaptability of populations (Du et al., 2016; Toro & Caballero, 2005). Many plant species are rich in diversity due to their wide distribution and a large number of individuals (Han et al., 2020). The study of their genetic variation is of great significance for the effective selection and maintenance of the diversity of germplasm resources (Belaj et al., 2012). Morphological traits have been applied to assess genetic variability, and the stability of results may be affected by environmental conditions. As an alternative, molecular markers are more stable and reliable for evaluating germplasm resources and have been used to evaluate the genetic variability of various species (Mafakheri et al., 2020; Rasul, Grundler & Tahir, 2022). Simple sequence repeats (SSRs) are the most frequently used molecular markers in population genetics. Due to the advantages of high polymorphism, specificity, reproducibility and comprehensive genome coverage, SSRs have been used to analyse population genetic diversity, structure and differentiation in species, such as *Cunninghamia lanceolata* (Hang et al., 2019), *Pinus massoniana* (Feng et al., 2014), *Populus tomentosa* (Du et al., 2012), and *Pinus sylvestris* (Pazouki et al., 2016).

The above research are all concerning diploids, but polyploidy is common in *C. hainanica*, mainly including octaploids and decaploids; however there are different reports of ploidy, such as on tetraploids, pentaploids, hexaploids and heptaloids (Ye et al., 2021). The uncertainty about polyploid inheritance patterns makes population genetic analysis more complex and challenging (Meirmans & Tienderen, 2013). For polyploid genetic analysis, a method has been developed to handle SSR data of polyploids for population genetic analysis (Dufresne et al., 2014), and the genetic diversity, structure and differentiation of polyploid plants were analysed using SSRs based on this method (Huang et al., 2018).

In this study, we used wild *C. hainanica* as material to determine the somatic chromosome count and the genetic structure and differentiation across different provenances by SSRs. The primary purpose of our study was to quantify the level of genetic diversity, and assess the genetic differentiation and structure of *C. hainanica* populations. Additionally, we provide support for the evaluation and utilization of genetic resources in *C. hainanica*.

## Materials and Methods

### Plant sampling

Wild *C. hainanica* samples were collected from major georeferenced sampling sites covering the entire longitude and latitude range of Hainan Province. The latitude and longitude of each sampling site were determined using a handheld GPS (GarmineTrex Handheld GPS; Garmin). We used ArcGIS (Esri) programs to draw a population distribution map based on population distribution coordinates. Plant individuals are randomly selected from each population, and the number of samples chosen depended on the size of the population. Ninety-six plant individuals from six wild *C. hainanica* populations were assigned. We collect fresh leaf material from each individual and stored it at −80 °C for DNA extraction.

### DNA extraction, amplification, and microsatellite genotyping

The DNeasy Plant Mini Kit (Tiangen Biotech Co. Ltd., Beijing, China) was used to extract the DNA of the leaf sample, and the operation of this application was strictly conducted according to the manufacturer’s instructions. Likewise, a NanoDrop 2000 spectrophotometer (Thermo Scientific, USA) and agarose gel assay were used to measure the quality of the DNA samples. Twelve highly polymorphic SSR markers were used to genotype 96 samples of *C. hainanica* (Table 1). The target DNA fragment was amplified using the fluorescently labelled TP-M13-SSR polymerase chain reaction (PCR) method (Schuelke, 2000). The universal M13 primers fluorescently labelled with 6-carboxy-x-rhodamine, 6-carboxy-fluorescein, tetramethyl-6-carboxyrhodamine, or 5-hexachloro-fluorescein and the forward primer with a universal M13 primer tail (5-TGTAAAACGACGGC CAGT-3) at the 5′ end were used for the previously described PCR method. The PCR procedure was as follows: denaturation for 2 min at 95 °C; 12 cycles of 30 s at 95 °C, 30 s at 64–59 °C (−0.5 °C per cycle), 1 min at 72 °C; then, 24 cycles of 30 s at 95 °C, 30 s at 65 °C, 1 min at 72 °C, and a extension of 2 min at 72 °C (Cui et al., 2021). PCR amplification was performed in a Thermal Cycler (Bio-Rad, Hercules, CA, USA), and an ABI 3730XL DNA Analyser (Applied Biosystems, USA) was used for the detection of PCR products. The fluorescence detection data from capillary electrophoresis were used for SSR analysis with the GeneScan™ 500 LIZ^^®^^ Size Standard as an internal standard, and allele analysis for each SSR locus was performed using GeneMapper version 3.7 (Applied Biosystems, Foster City, CA, USA).

**Table 1:** The primary simple sequence repeat primers used in the study.

Locus	Sequence (5′–3′)	Repetitive unit	Repetitive time	Annealing temperature (°C)	No. of Alleles (A)	PIC	SPI (PIC ×A)	Allele range size (bp)
Loc1	F: CCCAGCACCCAGAATCAGAA	CCT	6	66	7	0.810	5.67	180–201
	R: TCGTCTCTCAATTGCGCGAT							
loc2	F: GCGTATGGAAAAGCTGAGAA	TC	10	57	28	0.945	26.46	156–292
	R: GAAGCAAACCACTGAGGTGA							
Loc3	F: TTGTAGGCTGCCCAATCTCC	GAA	6	66	10	0.754	7.54	278–305
	R: CCATCTCAACCATCACCCCC							
Loc4	F: TCCGAAACCGTGCCTCATTT	GAA	5	65	15	0.828	12.42	243–310
	R: ATCAAGCCCGGTTGTTGTCA							
Loc5	F: GCCGTCTGTCTTCATCCGAA	CTC	5	66	9	0.711	6.399	170–294
	R: ACCACAGCTCAAACACACCA							
Loc6	F: TACCTCTCACAGCCTCCTCC	TCT	5	67	7	0.564	3.948	245–269
	R: CCCAACTACCCCCACTGTTC							
Loc7	F: GATCTGTGTCTCTCTGTTCCC	TG	32	55	9	0.760	6.84	187–215
	R: CCACACATCATCTTTTCCTC							
Loc8	F: GAAGTTTGTTGAGAGTGCTGC	TA	22	55	14	0.875	12.25	156–204
	R: ACAGATCTAAATTTGGGGGG							
Loc9	F: CTCACTACAGCRGCAACCGC	TC	11	55	9	0.731	6.579	170–260
	R: CCTGAATCTAGTGGGGCTTC							
Loc10	F: TCACACCAGCCCCAAAAAGT	CAC	5	65	17	0.857	14.569	174–309
	R: CTAGAAACCCCGCGAGTCTC							
Loc11	F: GCCGAAACCCAGGATCAGAA	CCG	5	66	10	0.788	7.88	203–293
	R: CTCAACCTCCTCAGCTGGTG							
Loc12	F: GATGACAGGCCTGCGAAATG	TG	8	59	8	0.705	5.64	204–350
	R: TCAACGAAGCATACACAACGT							
Mean			10.000	61.833	11.917	0.777		

### Chromosome counts and ploidy levels

The young leaves of *C. hainanica* were collected and placed in an ice box, and celllysates (Precise-P, Sysmex) and DAPI staining solution (4′,6-diamidino-2-phenylindole) were placed in an ice box to precool. Approximately 0.5 g of fresh leaf tissue was chopped using a sharp razor blade in a 55 mm plastic Petri dish containing 300 of µL celllysate and 10 µL of PVP aqueous solution (28.57%, mass fraction). The separation buffer containing the nuclei was filtered into a 1.5 mL centrifuge tube using a 40 µm nylon filter, and 200 µL of DAPI staining solution was immediately added. After incubation for 30 s in an ice bath, the fluorescence intensity of DAPI-stained nuclei was determined using a Partec CyFlow^^®^^ Space flow cytometer (Partec, Munich, Germany). Each DAPI-stained nuclei solution was tested for three replicates, and each repetition comprising 5000–10000 counts was measured. *Camellia fluviatilis* (2n =2x =30), a diploid relative species, was used as the control (Huang et al., 2018). Somatic cell chromosome counting was further validated using carbol fuchsin staining (Ye et al., 2020).

### Genetic analysis

The nonamplified (null) alleles at each microsatellite locus were detected using Micro-Checker v2.2.3 software (Van Oosterhout et al., 2004). The allele data from Genemarker were imported into POLYSAT version 1.4 (Clark & Jasieniuk, 2011), and the data were exported into the GenoDive format (Meirmans & Van Tienderen, 2004) to calculate the number of alleles, effective number of alleles, observed heterozygosity, expected heterozygosity and inbreeding coefficient for every population and every locus. In addition, Hardy-Weinberg equilibrium tests and various estimates of genetic differentiation between populations were performed using GenoDive version 2.0, and analysis of molecular variance (AMOVA) was performed to estimate sources of genetic variation between populations and within individuals (Meirmans, Liu & Van Tienderen, 2018a).

### Population structure

STRUCTURE version 2.3.3 (https://web.stanford.edu/group/pritchardlab/structure_software/release_versions/v2.3.3/html/structure.html) is more reliable than other clustering methods for cluster analysis of mixed ploidy populations (Stift, Kolar & Meirmans, 2019) and was used to estimate the *C. hainanica* population structure. Population genetic structure analysis based on the Bayesian clustering method was performed using an admixture model with independent allele frequencies (Meirmans & Liu, 2018b). We set the number of groups (K) from 1 to 6 and the length of the burn-in period of MCMC (Markov chain Monte Carlo) to 100,000 steps followed by 1,000,000 MCMC steps. Then, we calculated the lnP (D) value corresponding to each K value10 times, and calculate the average lnP (D). In addition, an ad hoc algorithm was used to calculate the K-1 ΔK value. Finally, the K value corresponding to the highest ΔK according to the fluctuation of all ΔK value peaks was the optimal number of subpopulations for the genetic structure of the population (Pritchard, Stephens & Donnelly, 2000; Evanno, Regnaut & Goudet, 2005). The bar charts for the data derived from the STRUCTURE analysis were created using DISTRUCT version 1.1 software packages (Rosenberg, 2003). Principal coordinate analysis (PCA) was performed using POLYSAT version 1.4 (Clark & Jasieniuk, 2011), and the genetic distance between samples was calculated using the Bruvo distance (Bruvo, Michiels & Schulenburg, 2004).

## Results

### Ploidy analysis

**Figure 1:**
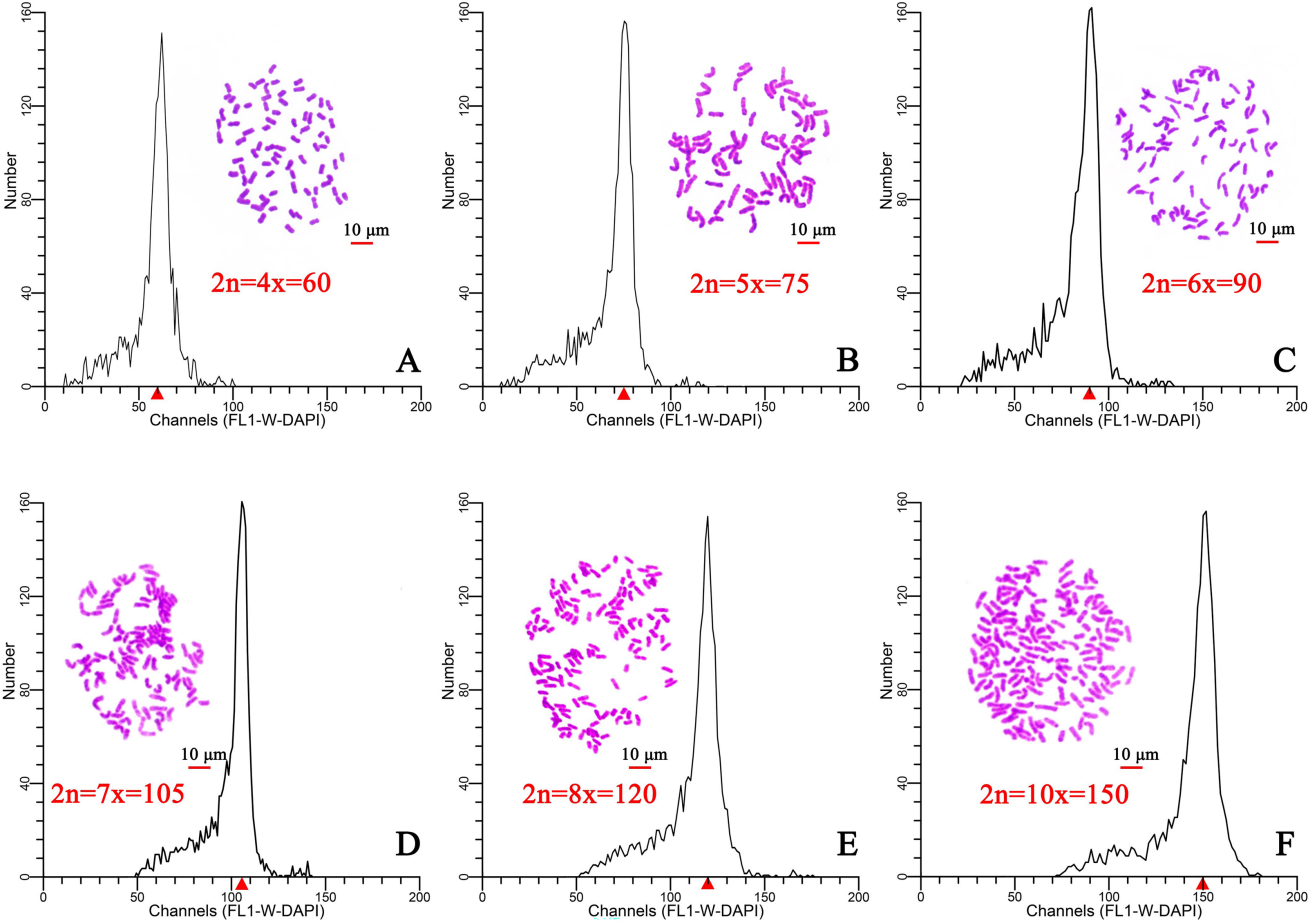
Chromosome counting and histograms presenting the flow cytometric analysis results, Scale bar =10 µm. (A) Tetraploid plants with 2n=4x=60; (B) pentaploid plants with 2n=5x=75; (C) hexaploid plants with 2n=6x=90; (D) heptaploid plants with 2n=7x=105; (E) octoploid plants with 2n=8x=120; (F) decaploid plants with 2n=10x=150. A triangle (Δ) represents the fluorescence intensity detected by flow cytometry.

Flow cytometry was used to determine the ploidy of 96 plants from six different regions in Hainan Province. After staining with DAPI (100 µL, 50 µg/mL), the relative DNA content of each plant was determined. The results indicated that the relative DNA content in hexaploid plants was 1.5-fold higher than that of in tetraploid plants (Figs. 1A, 1C). The ploidy was estimated at tetraploid, pentaploid, hexaploid, heptaploid, octoploid, and decaploid for all samples (Fig. 1). Moreover, a chromosome tabletting technique indicated that the tetraploid plants contained 60 chromosomes, the pentaploid plants contained 75 chromosomes (2n=5x=75) (Fig. 1B), the hexaploid plants contained 90 chromosomes (2n=6x=90) (Fig. 1C), the heptaploid plants contained 105 chromosomes (2n=7x=105) (Fig. 1D), the octoploid plants contained 120 chromosomes (2n=8x=120) (Fig. 1E), and the decaploid plants contained 150 chromosomes (2n=10x=150) (Fig. 1F). These results indicated that the ploidy of wild *C. hainanica* from different provenances in Hainan Province includes tetraploid, pentaploid, hexaploid, heptaploid, octoploid and decaploid, and that there is complex ploidy differentiation in the *C. hainanica* species.

### Sample assessment

**Figure 2:**
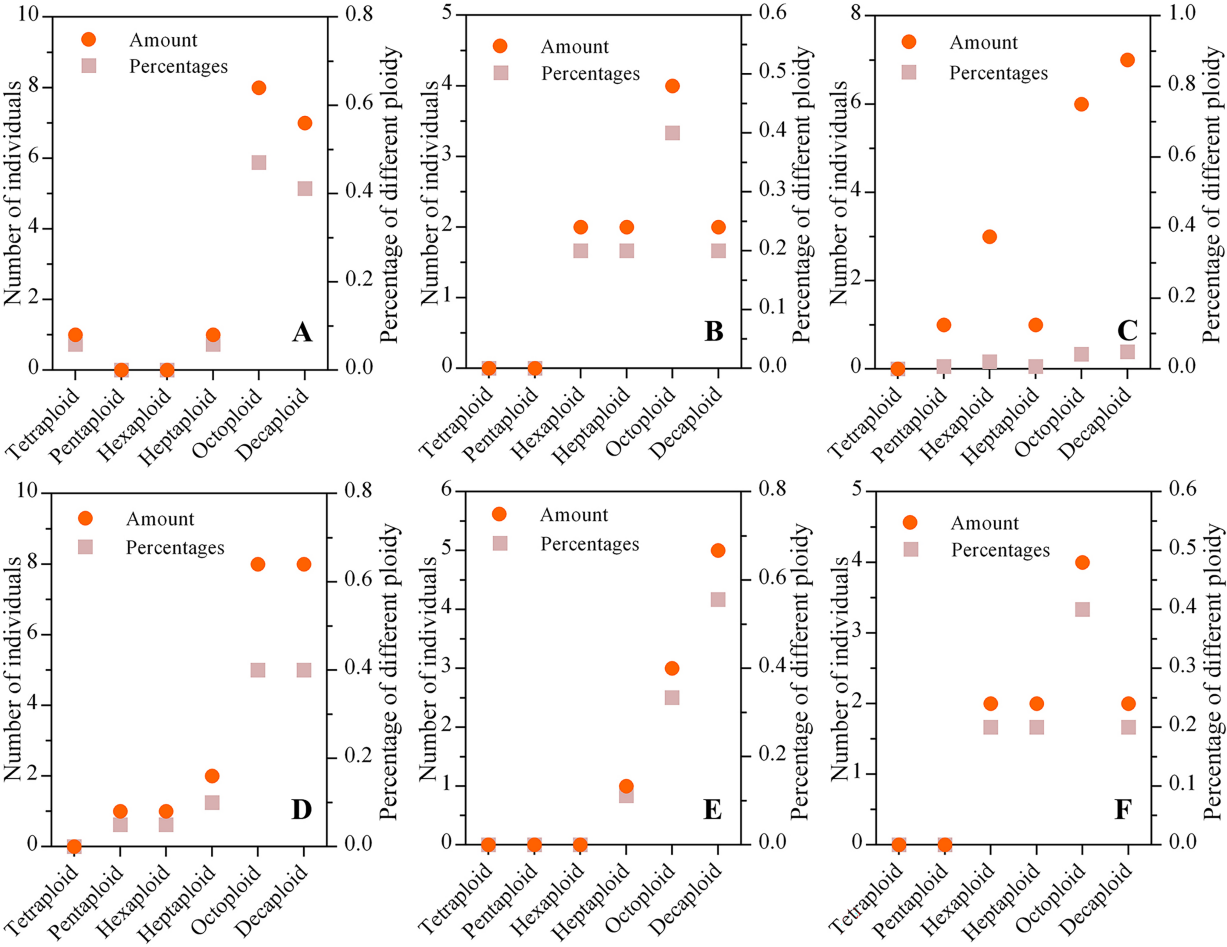
The ploidy composition of populations from different populations. (A) Tunchang; (B) Wuzhishan; (C) Qionghai; (D) Qiongzhong; (E) Chengmai; (F) Dingan; The ordinate on the left represents the number of individuals in each population, and the ordinate on the right represents the percentage of different ploidies in each population. The dot represents the number of individuals with different ploidies, and the square represents the proportion of varying ploidy plants in the population.

The mixed ploidies were recognized in a few populations *via* the ploidy test of every individual. We found that the numbers of tetraploid, pentaploid, hexaploid, heptaploid, octoploid and decaploid plants were 1, 2, 9, 13, 29, and 42, respectively, in six wild *C. hainanica* populations, which showed that the higher the ploidy was, the greater the number of plants. In addition, octaploids and decaploids accounted for approximately 74% of all samples, and the number of tetraploid, pentaploid, hexaploid and heptaploid plants was lower than that of octaploids and decaploids in each population (Fig. 2).

### Genetic diversity and inbreeding coefficient estimates of *C. hainanica*

**Figure 3:**
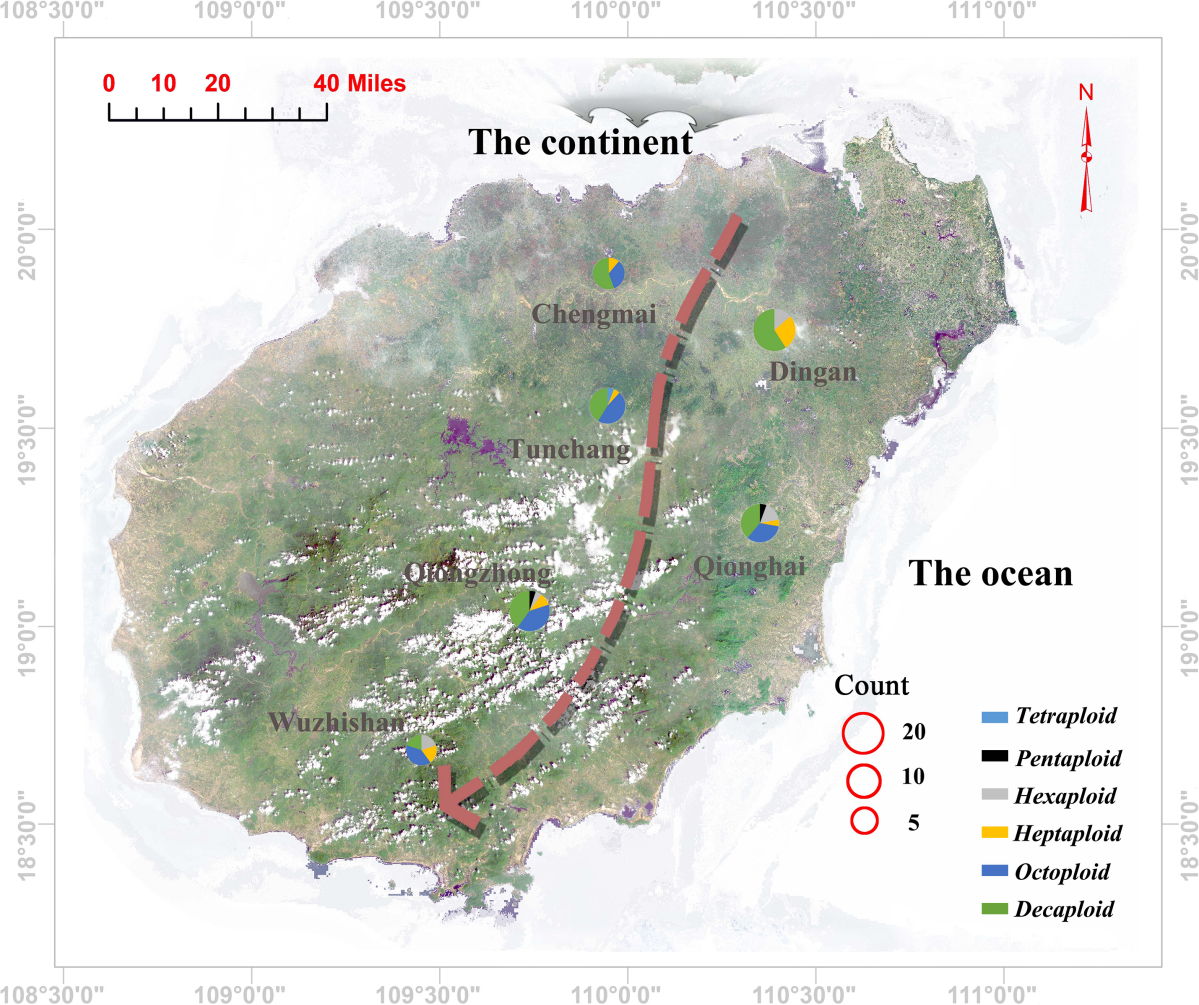
Geographic locations of the sampling sites and corresponding ploidy assignments (pie charts) for the 96 individuals in Hainan Province. Each pie chart is divided into several coloured sections according to the proportion of individuals of different ploidies collected in its population (blue: tetraploid; red: pentaploid; grey: hexaploid; yellow: heptaploid; purple: octoploid; green: decaploid); The size of the pie charts represents the number of selected individuals in each population; the red circles represent the size of the population. The arrow direction indicates a gradual decrease in the genetic diversity of the population.

High polymorphism SSR markers with a mean number of alleles equal to 7.931 and a mean effective number of alleles equal to 4.217 were used to estimate the genetic diversity and inbreeding coefficient. Low genetic diversity was found for the *C. hainanica* populations, and heterozygosity decreased with decreasing latitude from Chengmai, Dingan, Tunchang, Qionghai, Qiongzhong to Wuzhishan, as shown in the arrow direction (Fig. 3), while Gis values increased accordingly (Table 2). The Chengmai population had the highest genetic diversity estimates, and the observed heterozygosity and expected heterozygosity were 0.558 and 0.752, respectively. Meanwhile, the Wuzhishan population had the lowest genetic diversity estimates, and the observed heterozygosity and expected heterozygosity were 0.335 and 0.674, respectively. Positive inbreeding coefficient Gis values were found for the whole population, which showed significant loss of heterozygosity. However, the overall observed heterozygosity and expected heterozygosity were not similar for the same population, the observed heterozygosity was lower than the expected heterozygosity, and the genetic diversity estimates decreased with increasing Gis values accordingly.

### Genetic differentiation coefficient of *C. hainanica*

Population genetic differentiation analysis showed that the degree of genetic differentiation among populations was low (Fst<0.100) (Table 3). The coefficient of genetic differentiation was the lowest between the Chengmai and Dingan populations (Fst =0.016), while Fst was the highest between the Tunchang, Qionghai and Qiongzhong Wuzhishan populations (Fst =0.066, 0.064, 0.091). The results showed that there was moderate genetic differentiation among populations. The results of the AMOVA calculation showed that (Table 4), the genetic variation within each population accounted for 85% of the total genetic variation (*P* = 0.001). The genetic variation within a population is much higher than the genetic variation among populations.

### Genetic structure of *C. hainanica*

**Figure 4:**
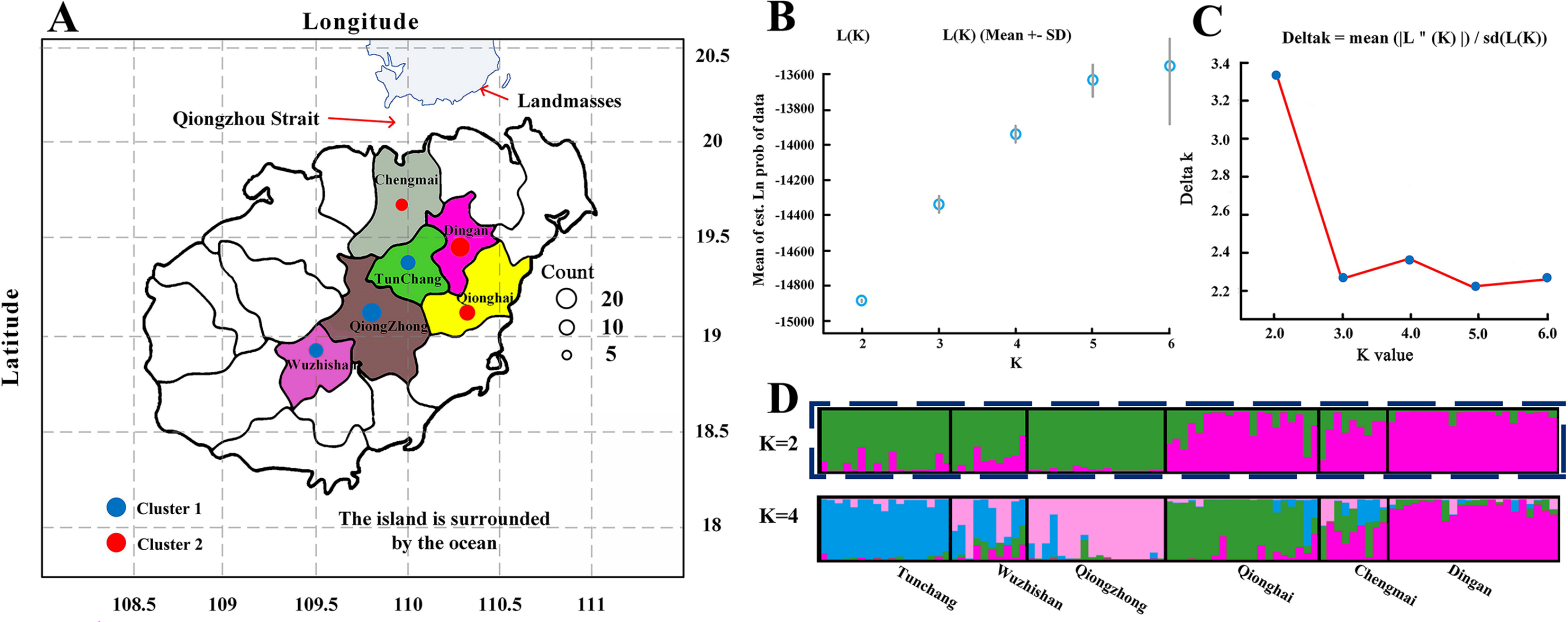
The distribution and genetic structure of 6 populations of *C. hainanica*. (A) The distribution of *C. hainanica* populations. The size of the circles represents the number of individuals in each population. The red pie chart represents Cluster 1 and the blue pie chart represents Cluster 2, when *K* = 2. Bayesian inference analysis for determining the most likely number of clusters (K) for the distribution of (B) the likelihood L(K) values and (C) Δ K values is presented for K = 2–6. (D) STRUCTURE plots are presented for *K* = 2 and *K* = 4. Each vertical bar represents a population and its assignment proportion into population clusters (K).

The *C. hainanica* populations come from different provenances (Fig. 4), and there may be subpopulations. Therefore, the genetic structure of these populations was estimated by STRUCTURE software. When the Bayesian clustering method was used to analyse the *C. hainanica* population, Ln Pr(K) increased gradually with increasing K value (Fig. 4B), and the optimal number of subpopulations could not be selected by this method. ΔK values corresponding to *K* = 1 − 6 were counted, and the ΔK statistic value reached the maximum value when *K* = 2 (Fig. 4C). Therefore, the individuals in all populations could be divided into 2 groups (Fig. 4A). The individuals from the Tunchang, Wuzhishan and Qiongzhong regions were divided into Cluster 1, while the individuals from the Qionghai, Chengmai and Dingan regions were divided into Cluster 2. We performed principal coordinate analysis (PCA). The results of PCA showed that *C. hainanica* samples from Tunchang, Wuzhishan and Qiongzhong were separate from those from Qionghai, Chengmai and Dingan, which verified the STRUCTURE analysis (Fig. S1). The results indicated clear genetic differentiation between the Tunchang, Wuzhishan, and Qiongzhong regions and the Qionghai, Chengmai, and Dingan regions. In addition, we found lower genetic differentiation between the Qionghai, Chengmai, and Dingan regions and the Tunchang, Wuzhishan, and Qiongzhong regions.

**Table 2:** Indices of genetic diversity *C. hainanica* population.

Population	Num	Eff_num	H_o_	H_e_	G_is_
Chengmai	7.167	5.128	0.558	0.752	0.240
Dingan	9.083	4.320	0.497	0.734	0.241
Tunchang	8.167	4.160	0.466	0.694	0.283
Qionghai	8.250	4.082	0.439	0.694	0.328
Qiongzhong	7.333	3.925	0.413	0.690	0.402
Wuzhishan	7.583	3.689	0.335	0.674	0.502
Overall	7.931	4.217	0.451	0.706	0.333

**Notes.**

Num Number of alleles Eff_num Effective number of alleles Ho Observed heterozygosity He Expected heterozygosity Gis Inbreeding coefficient

**Table 3:** The coefficient of genetic differentiation (*F*_st_) among populations.

Site	Dingan	Tunchang	Qionghai	Qiongzhong	Wuzhishan
Chengmai	0.016	0.030	0.044	0.037	0.064
Dingan		0.030	0.021	0.019	0.037
Tunchang			0.066	0.064	0.091
Qionghai				0.012	0.031
Qiongzhong					0.011

**Table 4:** Analysis of molecular variance (AMOVA) based on 12 SSR markers.

Source of variation	SSD	df	MS	VC	PV	F-value	*P*-value
Within population	351.038	90	3.9	3.9	0.85		
Among population	73.534	5	14.707	0.688	0.15	0.15	0.001

**Notes.**

df degrees of freedom PV percentage of variation SS sum of squares VC variance components

## Discussion

### Ploidy of *C. hainanica*

The flow cytometry and somatic chromosome count results indicated that wild *C. hainanica* samples had an inconsistent ploidy at different provenances, and the ploidy of the samples in the same provenance was different. Complex intraspecies multiploidy is common in *C. hainanica*, and previous research supports this conclusion (Ye et al., 2021). The reason may be the fusion of an undiminished gamete with a normal gamete (Zhang et al., 2021). The *C. hainanica* samples used in our study contained mixed-ploidy, which verified previous findings that there is complex intraspecific polyploidy in the genus *Camellia* (Zhuang, 2008). Polyploid species of the genus *Camellia* are more common in the western, northern, and northeastern regions, but are rarely found in the south (Ming, 2000). However, *C. hainanica* was found in Hainan Province in the southernmost region of China. Therefore, more samples from diverse distribution regions should be used to confirm the intraspecific polyploidy phenomenon.

### Genetic diversity of *C. hainanica*

The genetic diversity of a species reflects its evolutionary and adaptive potential. The more genetic variation a species has, the more adaptive it is Du et al. (2012). Therefore, the genetic diversity of species is necessary to be studied to define their biological properties (Zhang et al., 2019). Our study shows that *C. hainanica* populations have high levels of genetic diversity with a high average expected heterozygosity (0.706), although the expected heterozygosity is higher than the results of previous research for other *Camellia* species (Tang et al., 2006; Lin et al., 2013; Li et al., 2019), and the higher heterozygosity may be due to a recent polyploidy event. The high degree of population genetic variation for polyploid species increases the genetic diversity of polyploidy (Huang et al., 2013). Similar to the results of population genetic analyses by Cui et al. (2021) observed heterozygosity (H_o_ = 0.451) estimates were also lower than the expected heterozygosity (H_e_ = 0.706), indicating significant heterozygosity deficits in all populations. Meanwhile, we found that the average inbreeding coefficient was positive (G_is_ = 0.333) for all *C. hainanica* populations, and the level of inbreeding was higher, which may have resulted from geographical isolation between islands and continents.

### Genetic structure and genetic differentiation of *C. hainanica*

Differences in population genetic structure reflect genetic diversity, which conveys the adaptation potential of a species to its changing environment (Melo et al., 2014; Chen et al., 2021; Niu et al., 2019). In our study, *F*_st_ underestimated genetic differentiation between provenances, while lower genetic differentiation occurred among the six provenances tested. Meanwhile, the analysis of molecular variance (AMOVA) revealed that the genetic variation within the population accounted for 85% of the total genetic variation, which is consistent with the low *F*_ST_ values (Fst<0.100) among provenances. The results are similar to those of previous studies of the genus *Camellia*, and island isolation may play a key role in this genetic differentiation (Lin et al., 2013). In addition, the low degree of variation may be due to the relatively small geographic regions in our survey being from similar climatic provinces, which might be another factor resulting in low differentiation. Genetic structure analyses using both PCA and structure indicated that *C. hainanica* plants are genetically differentiated and showed that *C. hainanica* plants could be clustered into two clusters (Fig. 4). The two clusters were located in the northeast with the coastal area and southwest being a certain distance from the ocean, which matches the geographic provenance of the individual. As indicated by Huang et al. (2018), *C. hainanica* is insect pollinated and gene flow frequency decreases with increasing distance between plants. As a result, plants in close proximity are grouped together.

## Conclusions

The study of the genetic diversity, genetic differentiation and genetic structure of *C. hainanica* will be helpful for exploiting its germplasm resources*.* Population genetic characteristics of *C. hainanica* have been discovered using flow cytometry and SSR markers, and the results focus on (1) complex ploidy differentiation in *C. hainanica*; (2) low genetic diversity and genetic differentiation among populations are low; and (3) the analysis of genetic structures in different populations, showing that *C. hainanica* can be divided into two clusters. Finally, to maintain the genetic diversity of *C. hainanica* and improve its potential application value, we must implement a protection strategy and then protect populations with low genetic diversity from evolutionary threats.

##  Supplemental Information

10.7717/peerj.14756/supp-1 Supplemental Information 1 Allelic configuration of each sample at 12 SSR loci

10.7717/peerj.14756/supp-2 Supplemental Information 2 The PCA analysis for *hainanica* populations Red circles represent samples from Tunchang, Wuzhishan, Qiongzhong and green circles represent samples from Qionghai, Chengmai, Dingan.

## Additional Information and Declarations

### Data Availability

The following information was supplied regarding data availability: The raw measurements are available in the Supplemental Files.
