# Supplementary figures and images for: Genetic differentiation and genetic structure of mixed-ploidy Camellia hainanica populations
Source: PeerJ. 2023 Feb 22;11:e14756. doi: 10.7717/peerj.14756 (PMC9961093; doi:10.7717/peerj.14756)

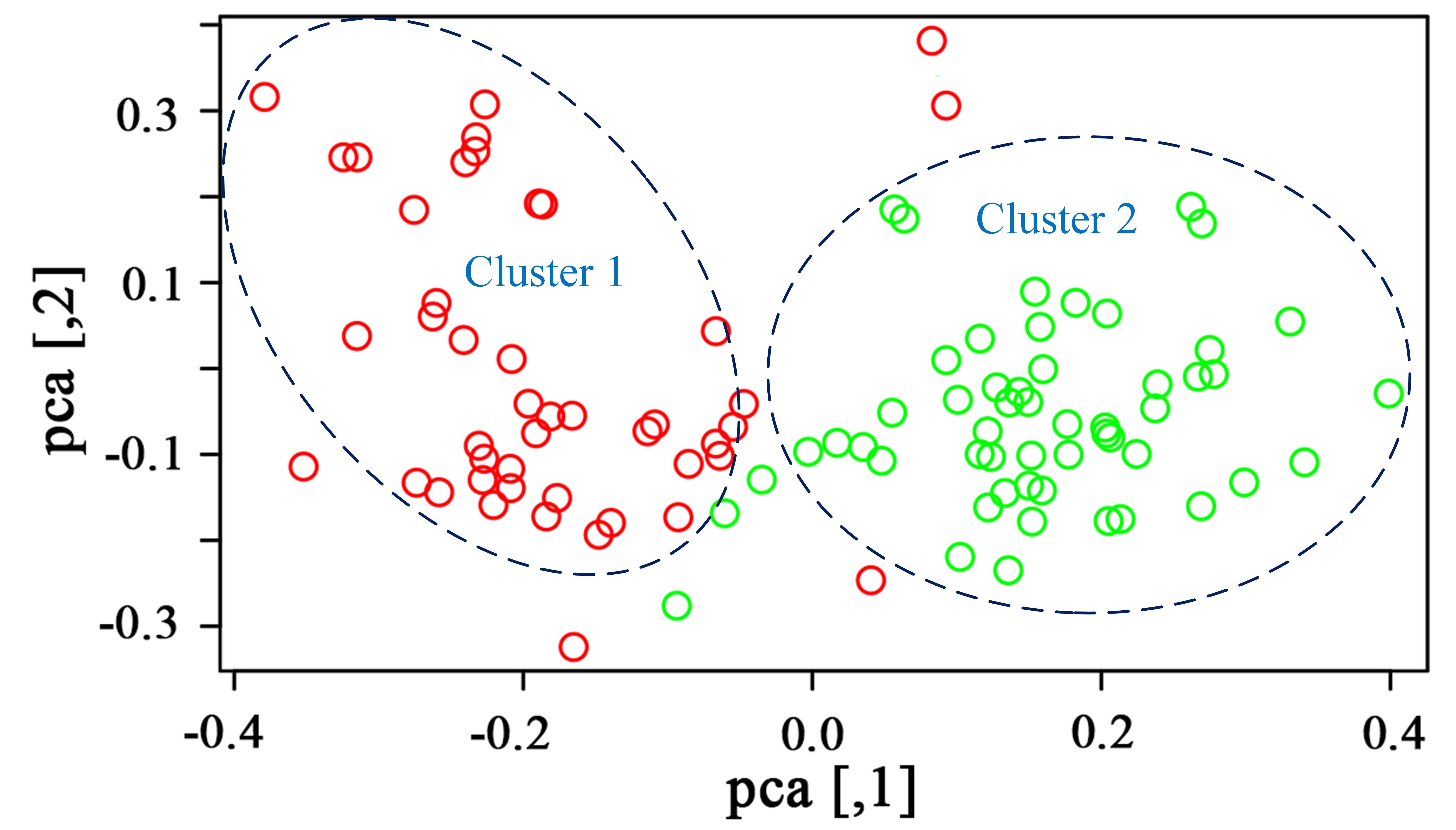

Supplement: Supplemental Information 2 — Red circles represent samples from Tunchang, Wuzhishan, Qiongzhong and green circles represent samples from Qionghai, Chengmai, Dingan. [file peerj-11-14756-s002.jpg]
